# Simulation of Fresnel Zone Plate Imaging Performance with Number of Zones

**DOI:** 10.3390/s20226649

**Published:** 2020-11-20

**Authors:** Ying Li, Ombeline de La Rochefoucauld, Philippe Zeitoun

**Affiliations:** 1 Laboratoire d’Optique Appliquée, CNRS, ENSTA-Paris, Institut Polytechnique de Paris, 828 Boulevard des Maréchaux, 91120 Palaiseau, France; ying.li@ensta-paris.fr; 2 Imagine Optic, Rue François Mitterrand, 33400 Talence, France; odlrochefoucauld@imagine-optic.com

**Keywords:** fresnel zone plate, modeling, wave propagator, X-ray integral imaging

## Abstract

In recent years, integral imaging, a promising three-dimensional imaging technology, has attracted more and more attention for its broad applications in robotics, computational vision, and medical diagnostics. In the visible spectrum, an integral imaging system can be easily implemented by inserting a micro-lens array between a image formation optic and a pixelated detector. By using a micro-Fresnel Zone Plate (FZP) array instead of the refractive lens array, the integral imaging system can be applied in X-ray. Due to micro-scale dimensions of FZP in the array and current manufacturing techniques, the number of zones of FZP is limited. This may have an important impact on the FZP imaging performance. The paper introduces a simulation method based on the scalar diffraction theory. With the aid of this method, the effect of the number of zones on the FZP imaging performance is numerically investigated, especially the case of very small number of zones. Results of several simulation of FZP imaging are presented and show the image can be formed by a FZP with a number of zones as low as 5. The paper aims at offering a numerical approach in order to facilitate the design of FZP for integral imaging.

## 1. Introduction

Integral imaging, a three-dimensional imaging approach first proposed by Lippmann in 1908 [1], has been developed rapidly in the past two decades with the advances both in micro-scale optical component and sensor fabrications, and in digital technology. The overall concept of integral imaging consists in capturing simultaneously the spatial and angular information of the light ray intersecting on the photosensor, the so-called 4D light field. The captured 4D light field allows us to refocus the image on different planes and render a 3D model of the sample with one single exposure [2,3]. The application of integral imaging in X-ray can become an important complement to X-ray computed tomography (CT). Nowadays, CT is one of the most common 3D imaging technique in clinical diagnosis, providing high accurate information of internal structure and guiding medical treatment. The principle of CT is rendering 3D image of the sample from multiple projections. It is apparent that large projection number will bring a better reconstruction quality [4]. Unfortunately, at the meantime it will deliver a high radiation dose for the patients and increase the probability of cancer [5,6]. Compared to CT, the X-ray integral imaging system has the prospect of reconstructing 3D images from one capture, and consequently leads to a lower dose and a short exposure time.

Various integral imaging acquisition systems have been proposed, such as camera arrays [7], micro-lens arrays [8], and amplitude masks [9]. For visible light, the design of placing a micro-lens array (MLA) in the front of conventional camera’s photosensor is widespread, in view of the diversity and accessibility of micro-lens array. However, the refractive index is very close to one for all materials at X-ray wavelengths, leading to a extremely long focal distances at kilometer scale for a single lens and a low numerical aperture ([10] pp. 66–71). Therefore, for the application on X-ray, we must search alternative optical components for the MLA.

The Fresnel Zone Plate (FZP), relying on the diffraction to realize X-ray focusing, is widely used and has been demonstrated to achieve a high spatial resolution [11]. The FZP array is thus available alternative to refractive lens array in X-ray imaging [12,13,14]. FZP imaging performance is dominated by its geometry; therefore, its number of zones is one of the most important structural parameters. For a desired focus, the larger the number of zones is, the higher resolution FZP can attain. Yet, due to micro-scale dimensions of FZP in the array of integral imaging system and current manufacturing techniques, the number of zones of FZP is limited.

Therefore, this paper attempts to numerically investigate the impact of FZP number of zones on its imaging performance, especially the case of very small number of zones. Accordingly, a simulation method based the scalar diffraction theory is introduced in this paper. Several simulation results of FZP imaging are presented and discussed, then compared to ideal refractive lens.

## 2. Method

**Figure 1:**
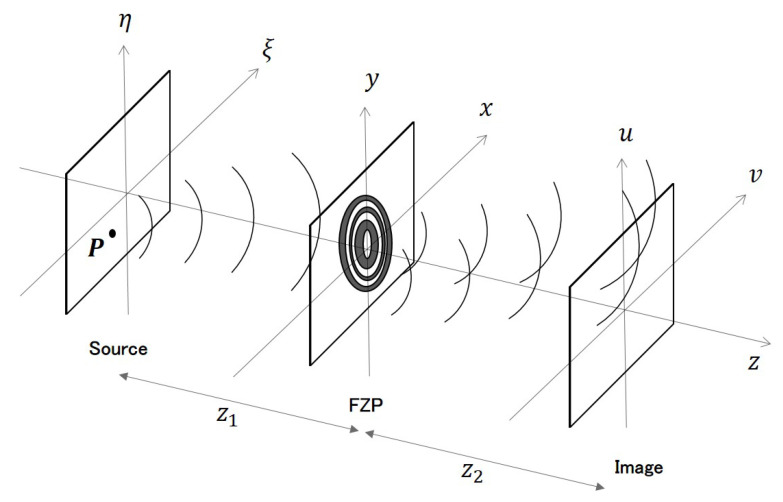
Generalized schematic of Fresnel Zone Plate (FZP) optical system: The point source P on the plane (ξ,η) emitting a spherical wave is intercepted and diffracted by the FZP on the plane (x,y). The image of the source is formed on the plane (u,v). $z_{1}$ and $z_{2}$ represent respectively the source–FZP distance and the FZP–image distance.

A Fresnel zone plate is a diffractive optic, its alternating transparent and opaque zones make the incident light constructively interfere at the desired focus. The FZP’s focusing properties can be analyzed by the scalar diffraction theory. Figure 1 represents a generalized schematic of a FZP optical system.

Considering a point source P(ξ,η), whose wave field distribution at a distance $\vec{r}$ is given by

(1) $$U_{0}=\frac{e^{i\vec{k}\cdot \vec{r}}}{\vec{r}}$$

Assuming the point source illuminates the entire FZP at distance $z_{1}$ on the plane (x,y), with the paraxial approximation and neglecting a pure phase factor, the incoming wave field $U_{zp}$ at the plane of FZP becomes

(2) $$U_{zp}=\frac{1}{i\lambda z_{1}}\exp\{\frac{ik}{2z_{1}}\left[{\left(x-\xi \right)}^{2}+{\left(y-\eta \right)}^{2}\right]\}$$

After passing through the FZP, the new wave field $U_{zp}^{\prime }$ distribution can be written as

(3) $$U_{zp}^{\prime }=t\left(x,y\right)\times U_{zp}$$

where t(x,y) is the transmission function of the FZP.

Then according to Huygens–Fresnel principle, at a distance $z_{2}$ behind the FZP, the wave field distribution at the image plane (u,v) is given by

(4) $$U_{zp}=\frac{1}{i\lambda z_{2}}\;\int \int \;U_{zp}^{\prime }\times \exp\{\frac{ik}{2z_{2}}\left[{\left(u-x\right)}^{2}+{\left(v-y\right)}^{2}\right]\}\;dxdy$$

On account of the linearity of the optical system [15], we can find the impulse response h(ξ,η,u,v) of the FZP, also known as the Point Spread Function (PSF), by setting ξ=η=0 in the above expression. The PSF is thus expressed as

(5) $$\begin{array}{cc} h\left(0,0,u,v\right)= & \frac{z_{2}}{z_{1}}\exp\left[\frac{ik}{2z_{2}}\left(u^{2}+v^{2}\right)\right]\\ & \times \;\int \int \;t\left(x,y\right)\exp[\frac{ik}{2}(\frac{1}{z_{1}}+\frac{1}{z_{2}})\left(x^{2}+y^{2}\right)]\exp\left[-\frac{ik}{z_{2}}\left(ux+yv\right)\right]\;dxdy \end{array}$$

The integral term of the impulse response expression can be considered as a Fourier transform with appropriate substitution of variables, yielding

(6) $$\begin{array}{cc} h\left(0,0,u,v\right)= & \frac{z_{2}}{z_{1}}\exp\left[\frac{ik}{2z_{2}}\left(u^{2}+v^{2}\right)\right]\\ & \times F\{t\left(\lambda z_{2}x^{\prime },\lambda z_{2}y^{\prime }\right)\exp\{\frac{ik}{2}(\frac{1}{z_{1}}+\frac{1}{z_{2}})[{\left(\lambda z_{2}x^{\prime }\right)}^{2}+{\left(\lambda z_{2}y^{\prime }\right)}^{2}]\}\} \end{array}$$

with $x^{\prime }=\frac{x}{\lambda z_{2}}$, $y^{\prime }=\frac{y}{\lambda z_{2}}$.

The transformation of the expression in the previous step allows performing an efficient numerical calculation with the help of Fast Fourier Transform (FFT) algorithm. Finally, the image Img(u,v) can be obtained by the convolution of the image predicted by geometrical optics img(u,v) with the impulse response h(ξ,η,u,v) [16]. It is worth noting that the application of the PSF on an image depends on the degree of coherence of the source. At the moment, we only consider the fully incoherent cases:

(7) $$Img\left(u,v\right)={\left|h\left(\xi ,\eta ,u,v\right)\right|}^{2}⊗{\left|img\left(u,v\right)\right|}^{2}$$

According to the convolution theorem, Equation (7) can be further converted to

(8) $$Img\left(u,v\right)=F^{-1}{\left\{F\{|h\left(\xi ,\eta ,u,v\right)\right|}^{2}{\}\times F\{|img\left(u,v\right)|}^{2}\}\}$$

This conversion reduces the complexity of the computation and allows greatly increasing the computation speed thanks to the use of FFT.

## 3. Results

The proposed method is flexible for the definition of FZP and the adjustment of imaging system structure, allowing to study the effects of different parameters on the FZP imaging performance. For the following part, the energy of source is set to 11keV. Several examples were performed and discussed in this section in order to validate the simulation approach and, in particular, to explore the impact of the FZP number of zones.

### 3.1. FZP Multiple Foci Imaging

A FZP has more than one diffraction orders, noted *m*, generating multiple focal spots. The focal length corresponding to the different orders is given by

(9) $$f_{m}=\frac{f}{m}$$

for diffractive orders m=0,±1,±3,±5… and *f* is the first order focal length ([10] pp. 337–348).

This imaging characteristic of FZP can be well simulated by the method previously described. We simulate a point source situated at a distance of 100f to approximate the parallel beam incident on a thin FZP. The transmission function of the FZP can be defined as

(10) $$t\left(r\right)=\left\{\begin{array}{c} 1,\;r_{n}<r<r_{n+1}\\ 0,\;else \end{array}\right.$$

where the $n_{th}$ zone radius $r_{n}\approx \sqrt{nf\lambda }$, λ is the wavelength of the source and *n* is the zone sequence number.

**Figure 2:**
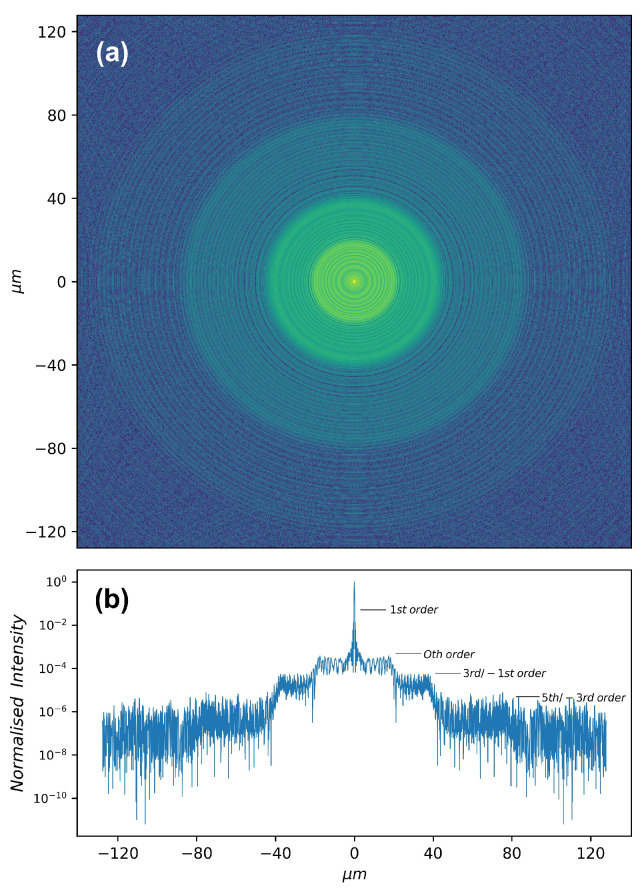
(**a**) False color image in logarithmic scale of the intensity distribution at the first focal plane of a FZP. The parameters of the FZP are focal length f=8.98 cm, radius r=20 μm, and number of zones N=40. The point source was set at an energy of 11 keV and situated at 100f prior the FZP. (**b**) Central row intensity profile of panel (**a**): the radii of 0th order, 3rd/−1st order, and 5th/−3rd order are, respectively, 20 μm, 40 μm, and 80 μm.

The simulated PSF at *f* is shown in Figure 2. Several orders appear: the +1st order focal spot, the 0th order undiffracted beam, the 3rd-order overlapped with the −1st order, etc. By the similar triangles and the Equation (9), the radius $R_{m}$ of each order circle at *f* is given by

(11) $$R_{m}=\left\{\begin{array}{c} (m-1)R,\;m>0\\ (1-m)R,\;m\leq 0 \end{array}\right.$$

where *R* is the radius of FZP.

Except the 1st order focal spot, the ratio of radii between 0th, 3rd/−1st, and 5th/−3rd order measured from the Figure 2 is 1:2:4. The measured ratio of radii between different orders agrees well with that calculated from Equation (11).

**Figure 3:**
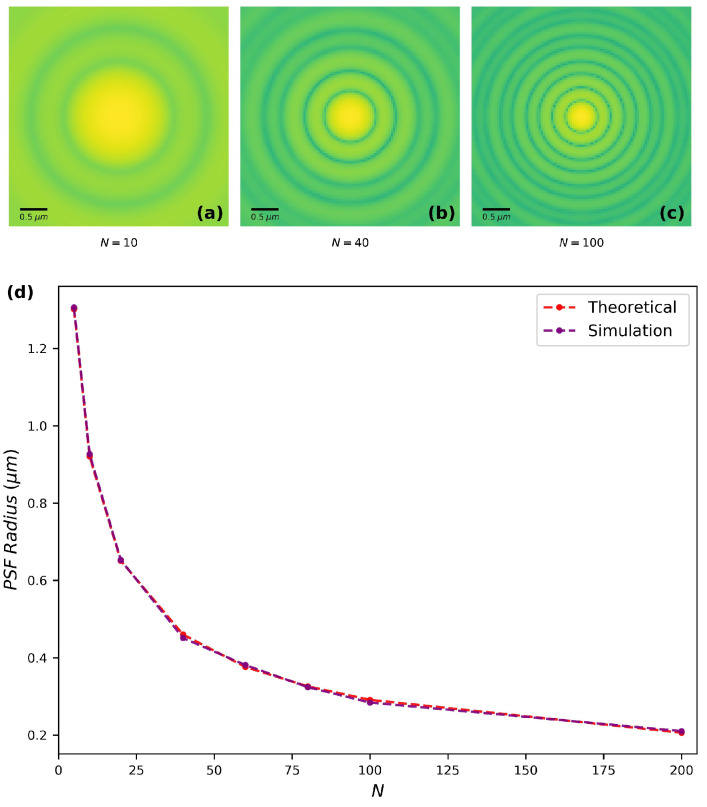
(**a**–**c**) False color images of simulated Point Spread Function (PSF) on a logarithmic scale, when the number of zones *N* equals to 10, 40, and 100. (**d**) Theoretical and simulated PSF spot radius versus N.

Further tests have been achieved by comparing the output of our model with theoretically calculated PSF of the FZP with different number of zones from 5 to 200 (Figure 3). For this calculation, a point source that emits a pure spherical wave is placed at 3f from the FZP. Three examples of numerical outputs with different number of zones *N* are displayed on Figure 3a–c. The variation of the PSF spot radius (the distance between the central maximum and the first minimum) versus the number of zones *N* is displayed in Figure 3d. When *N* increases, the PSF spot radius decreases. The theoretical spot radius calculated by the classical formula set by the Rayleigh criterion [17] (see Equation (12)) is reported and compared to the numerical results.

(12) $$theoretical\;spot\;radius=\frac{1.22\lambda }{D}\times z_{2}$$

where *D* is the FZP diameter and $z_{2}$ is the distance between the FZP and the image plane.

In general, the numerical results agree very well with theory, with a error within 2.4%. The errors are introduced not only from measurement bias, but also from the application of FFT in computational implementation. The errors and accuracy of numerical simulation due to the discrete sampling of FFT have been discussed in more detail in [18,19].

### 3.2. Variation of Detection Distance

**Figure 4:**
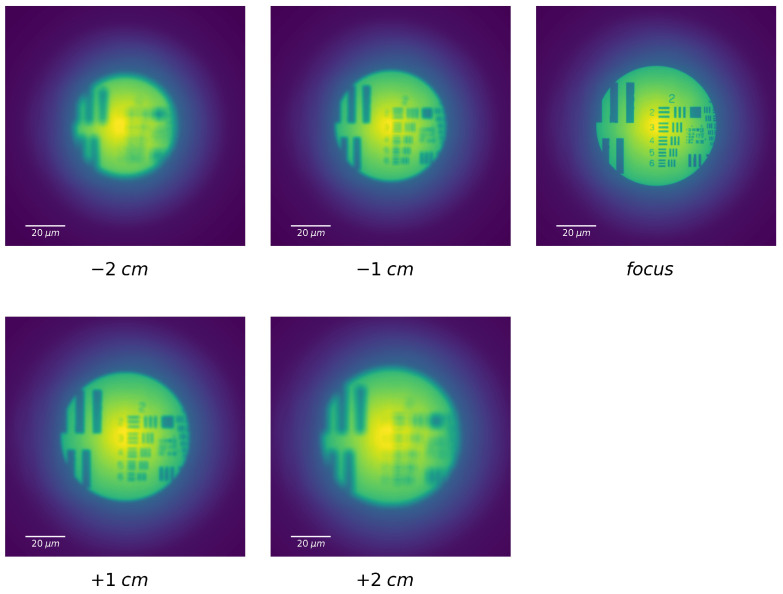
False color numerical images of a USAF 1951 target are recorded at various distances from the image plane in focus (called focus). The recorded distance is referred to the focal plane under each image. The focus is taken as the origin, and the negative and positive signs, respectively, represent the directions closer and further to the FZP. The FZP has the same parameters for Figure 2. The object to FZP distance is 3f and leading to the creation of an image on focus is at 1.5f. Each image dimension is 120×120 μm${}^{2}$.

After these conclusive tests of our numerical model, we performed optical simulation of the full model by imaging a classical target. Figure 4 displays a series of numerical images of the 1951 USAF resolution test chart before, at, and after the image plane. These numerical images are formed by the FZP with the same parameters as in Figure 2. The object to FZP distance is 3f, leading to the creation of an image at 1.5f. From Figure 4, we can clearly see the focusing process of FZP along the distance, i.e., the improvement of the image contrast and its worsening, as well as the halo around the 1st order image caused by the other diffraction orders. We may also observe the expected increase of the image size for increasing FZP–detector distance.

### 3.3. Lateral Resolution of Number of Zones on the Image Formation

**Figure 5:**
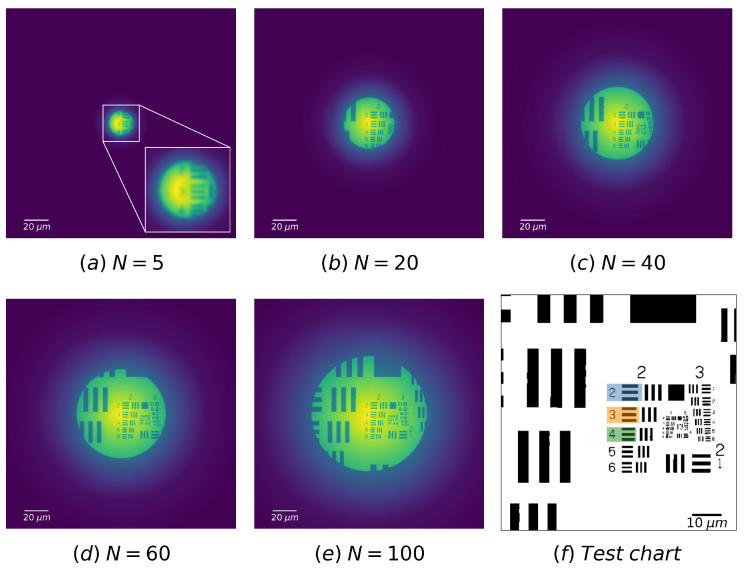
(**a**–**e**) False color numerical images of the USAF 1951 test target imaged by FZP with different number of zones. The object–-FZP distance is 3f and the FZP–image distance is 1.5f. Each image dimension is 200×200 μm${}^{2}$. (**f**) Zoomed part of interest on the test chart: for the following part, the blue, orange and green colored elements of bars in group 2 are respectively noted as elements 2.2, 2.3, and 2.4.

To our best knowledge, the quality of the image formed by a FZP versus the number of zones has never been studied. In order to perform this study, a set of incoherent images of the test chart were simulated for various numbers of zone *N*. The numerical images are carried out with the same FZP as well as the same geometry as for Figure 4. The results are displayed in Figure 5a–e and the part of interest on the test chart is zoomed in Figure 5f. It is apparent from Figure 5a–e that as *N* increases, the image becomes sharper. As the radius of FZP is meanwhile broadened, the field of view is enlarged.

**Figure 6:**
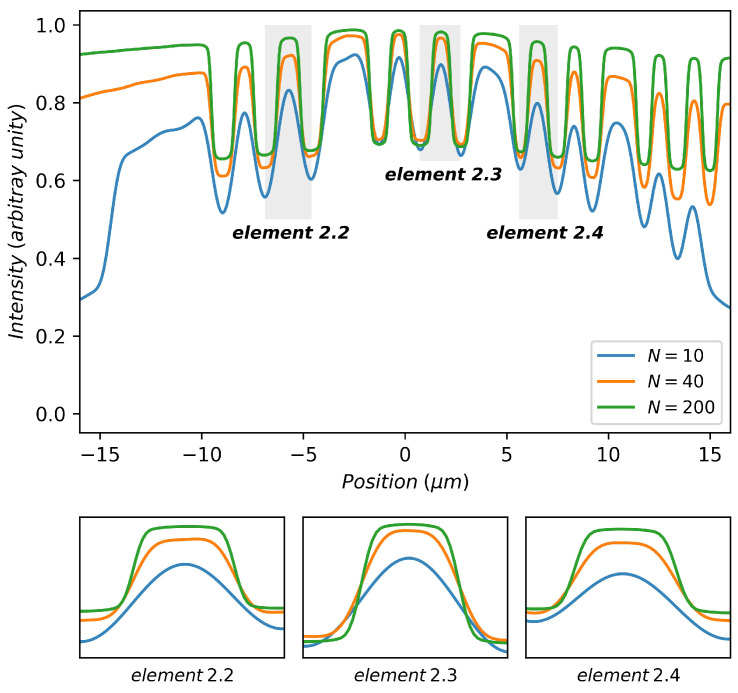
Intensity profile plots of images in focus with various numbers of zones: the images are simulated under the same conditions of Figure 4. Three zoomed parts of plot corresponding to different groups of bars are given below the main plot.

The variation of image quality with the number of zones is more evident in the intensity profile plot of bars. For example, the intensity profile of three groups of bars on the test chart (the blue-, orange-, and green-colored parts in Figure 5f are, respectively, named as elements 2.2, 2.3, and 2.4 in the following text) are plotted in Figure 6, varying the number of zones *N*. For the ease of comparison of the profile shape, the intensity plots have been normalized. While *N* increases, the plot shape of a bar approaches to a rectangle, which indicates the decrease in the PSF spot size (see in the inserts of Figure 6).

### 3.4. Quantification of the Effect of Number of Zones on the Image Formation

In order to quantify the evolution of the image quality versus the number of zones, several criteria might be use. One of the classical criteria, the contrast is chosen in this paper. It is defined as

(13) $$Contrast=\frac{I_{max}-I_{min}}{I_{max}+I_{min}}$$

with $I_{max}$ and $I_{min}$ representing the highest and lowest intensities in a studied zone.

**Figure 7:**
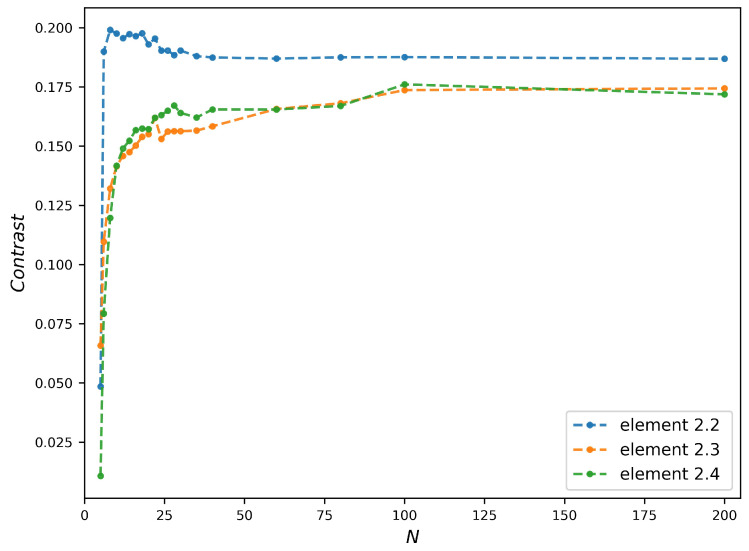
Contrast plots of different width bars versus number of zones *N*:the images are simulated under the same conditions of Figure 4, the studied bars of test chart are shown in Figure 4f.

The contrast of three sets of bars on the image of USAF1951 target (see in Figure 5f), respectively, with the width 1.36 μm, 1.18 μm, and 0.99 μm on the image plane were measured in the numerical images for different *N*. The results are plotted in the Figure 7. The contrast plots increase very quickly for the small *N* then evolve slowly. The trend of the contrast plots is consistent with the variation of the theoretical resolution of FZP with *N*. Moreover, the stabilization starting points vary with the width of bars.

Compared to Figure 3d, the contrast plots reach stabilization with a larger *N* than that corresponding to the needed PSF spot size (equal to the width of bar). Taking the bars of Element 2.3 as an example, the PSF spot size is already below the bars width when N=7, but the contrast plot roughly reaches stabilization at N=27. This delay of *N* is due to the fact that the stabilization of contrast requires the complete separation of points without overlap, which is stricter than the Rayleigh criterion used in Equation (12). Another remark in Figure 7 is the contrast value, whose maximum is as low as 0.2. In order to study its origin, we modeled the image formation with a theoretical perfect refractive lens. Supposed the FZPs in Figure 7 can be replaced by the refractive lenses with the same numerical aperture (NA), the transmission of refractive lens $t_{Lens}\left(x,y\right)$ is defined as

(14) $$t_{lens}\left(x,y\right)=\left\{\begin{array}{c} \exp\left[-\frac{ik}{2f}\left(x^{2}+y^{2}\right)\right],\;\sqrt{x^{2}+y^{2}}<R\\ 0,\;else \end{array}\right.$$

where *R* and *f* are the radius and focal length of lens, respectively.

**Figure 8:**
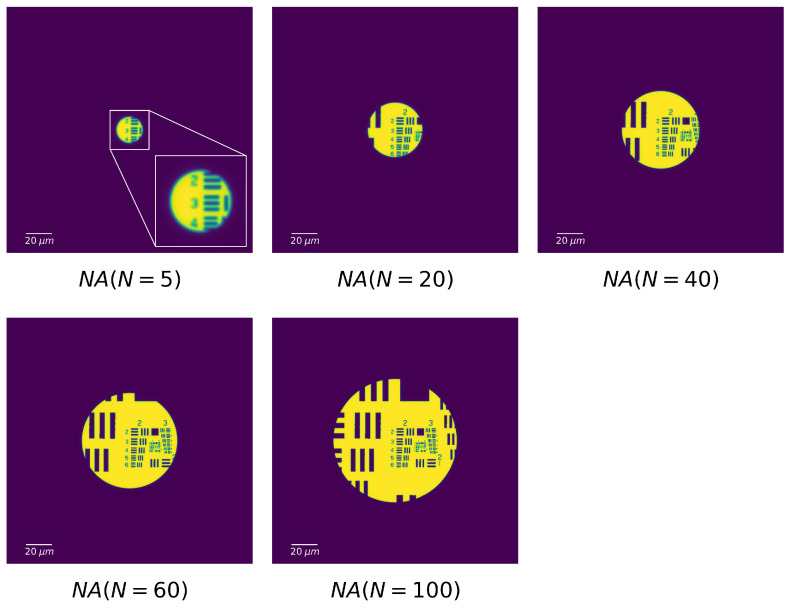
False color numerical images of the USAF 1951 test target imaged by refractive lens. The NA of the refractive lens equals to the NA of the FZP when the number of zones of FZP is 5, 20, 40, 60, and 100. Each image dimension is 200×200 μm${}^{2}$.

**Figure 9:**
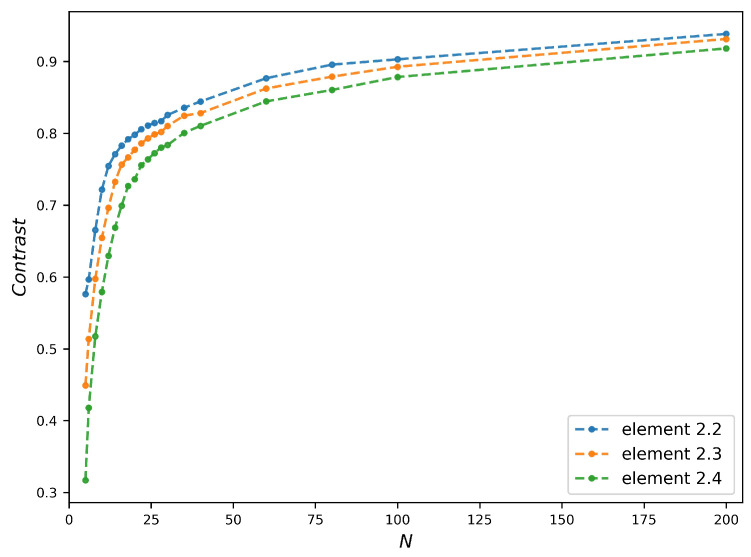
Contrast plots measured from the images formed by refractive lens: For the sake of clarity, the horizontal axis displays the number of zones of the FZP having the same NA than the refractive lens. Moreover, the images are simulated under the same conditions as for Figure 5.

A group of images formed by the refractive lens is simulated under the same structure of Figure 7, displayed in Figure 8. For the ease of comparison with Figure 7, the NA of refractive lens is written as a function of the number of zones. The contrast value measured from refractive lens images can reach a maximum of 0.94, much higher than that of FZP images (see in Figure 9). Moreover, the contrast plots of refractive lens are more continuous and smoother. This is because the first order image of FZP overlaps with the other orders, resulting in a brightened and complex background.

## 4. Conclusions

The paper aims at offering a numerical approach in order to facilitate the design of FZP for a integral imaging system in X-ray. In this paper, a simulation method based on the scalar diffraction has been introduced. The presented method is able to simulate the images formed by FZP with different parameters and at different position. The effect of the number of zones on image formation has been discussed, especially the case of very small number of zones. The results has shown that surprisingly image can be formed with a number of zones as low as 5. However, the usable contrast starts around 20 zones. In practice, experimental devices such as center beam stopper, order sorting aperture and Kohler illumination systems are commonly used to remove orders other than the 1st one of FZP. Therefore, the contrast can be further enhanced than the simulation, allowing the FZP to be used with very small number of zones. Future study can be extended to the axial effect of the number of zones, the formation of images in the case of partially to fully coherent beam and the application on the full set-up of integral imaging system.
